# Developing and evaluating non-invasive healthcare technologies for a group of female participants from a socioeconomically disadvantaged area

**DOI:** 10.1038/s41598-021-03262-3

**Published:** 2021-12-13

**Authors:** Eman Awad, Rathi Ramji, Stefan Cirovic, Margareta Rämgård, Anders Kottorp, Sergey Shleev

**Affiliations:** 1 Department of Biomedical Science, Health and Society and Biofilms-Research Center for Biointerfaces, Malmö University, 20560 Malmö, Sweden; 2 Department of Care Science, Health and Society, Malmö University, 20560 Malmö, Sweden

**Keywords:** Health care, Medical research

## Abstract

When compared to the general population, socioeconomically disadvantaged communities frequently experience compromised health. Monitoring the divide is challenging since standardized biomedical tests are linguistically and culturally inappropriate. The aim of this study was to develop and test a unique mobile biomedical testbed based on non-invasive analysis, as well as to explore the relationships between the objective health measures and subjective health outcomes, as evaluated with the World Health Organization Quality of Life survey. The testbed was evaluated in a socioeconomically disadvantaged neighborhood in Malmö, which has been listed as one of the twelve most vulnerable districts in Sweden. The study revealed that compared to conventional protocols the less intrusive biomedical approach was highly appreciated by the participants. Surprisingly, the collected biomedical data illustrated that the apparent health of the participants from the ethnically diverse low-income neighborhood was comparable to the general Swedish population. Statistically significant correlations between perceived health and biomedical data were disclosed, even though the dependences found were complex, and recognition of the manifest complexity needs to be included in further research. Our results validate the potential of non-invasive technologies in combination with advanced statistical analysis, especially when combined with linguistically and culturally appropriate healthcare methodologies, allowing participants to appreciate the significance of the different parameters to evaluate and monitor aspects of health.

## Introduction

Non-invasive healthcare technologies are an important part of research and development nowadays due to their low cost and convenience they offer to both healthcare receivers and providers^1^. In addition to traditional non-invasive technologies, that have been used for ages, *e.g.*, electrocardiography^2,3^, many other non-invasive appliances have been developed, such as cardiovascular diagnostic systems^4^, bioimpedance based scales^5,6^, and even non-invasive blood analyzers to measure not only physical parameters of the body, but also sentinel chemicals in blood, *e.g.*, hemoglobin (Hb)^7^, oxygen^8^, and glucose (Glu)^9,10^. In terms of precision, reproducibility, and reliability, some non-invasive chemical blood analyzers, *e.g.* glucometers, are less than adequate^11^, while pulse-oximeters are widely used in acute and critical care^12^. However, drawing on the obvious advantages, when developed further, non-invasive chemical blood analyzers might be considered as powerful tools for subgroups with special healthcare needs, such as infants and the elderly, as well as socially disadvantaged populations. These subgroups frequently experience subpar healthcare and compromised health in comparison to the majority population, the former owing to cognitive and communication issues, and the latter owning to inadequate living conditions. Socioeconomically disadvantaged populations often face challenges, such as social exclusion, discrimination, and poverty. They are also at a higher risk for mental health issues, as well as poor physical health status, *e.g*., suffering from obesity and hypertension due to poverty related malnutrition^13^. However, serious mental blocks, mainly distrust, to assess health in underserved areas using conventional protocols are also firmly established^14^.

Lindängen is a socioeconomically disadvantaged district in Malmö city, located in the South of Sweden. The Swedish Intelligent Unit has listed Lindängen as one of twelve vulnerable neighborhoods in the country. A recently published report reflects the challenges in Lindängen, *i.e.*, high crime rate, low education levels, high unemployment rates, and poor overall health^15^. The study reported herein has been conducted as part of a large program ”Collaborative Innovations for Health Promotion”^16^, which is based on community-based participatory research (CBPR)^17^ and aims to promote health among residents in Lindängen. An important aspect in the CBPR approach is to involve the participants throughout the various stages of the research process by way of dialogue and reflection, which also includes joint work with participants in the data gathering process to evaluate health outcomes^18^. Initially, the dialogue with the residents of Lindängen revealed that lack of access to low-cost health care was a contentious issue, eventually denying them the possibility to assess and understand their own physical health status^19^. Acknowledging the cost-efficiency of non-invasive healthcare technologies, along with minimal user discomfort and limited corporal interactions, it was decided to map the physical health of participants, reliant on non-invasive healthcare technologies. Apart from the obvious and immediate project outcome, *i.e*., the health map, user appreciation of the physical and social aspects of non-invasive healthcare technologies could be fully explored, predicting user compliance when choosing between alternative healthcare approaches.

The development of health promoting interventions among vulnerable populations is crucial in society. In order to improve health with various health interventions, it is important to incorporate objective as well as subjective health outcomes to monitor and measure various aspects related to a broad definition of health. The methods for measuring objective physical health should also match the specific needs among the target population^20^. Hence, rather than getting referrals from physicians to clinics in the established health care system, less intrusive and cost-effective health testing procedures should be exploited in socioeconomically disadvantaged areas, along with ready access to testbeds located in the immediate neighborhood. Thus, the aims of this research study were therefore threefold: first, to design a unique non-invasive portable testbed; second, to evaluate the physical health of the participants from the socioeconomically disadvantaged area; third, to explore the relationships between the non-invasive objective physical health measures and the self-perceived health, here expressed in Quality of Life (QoL). The third research question is crucial for exploration of the concept of health among the socioeconomically disadvantaged population, which is a multidimensional construct, viz*.*, a state of complete physical (objective) and mental/social well-being (subjective)^21^.

## Methods

### Development of biomedical testbed

**Figure 1:**
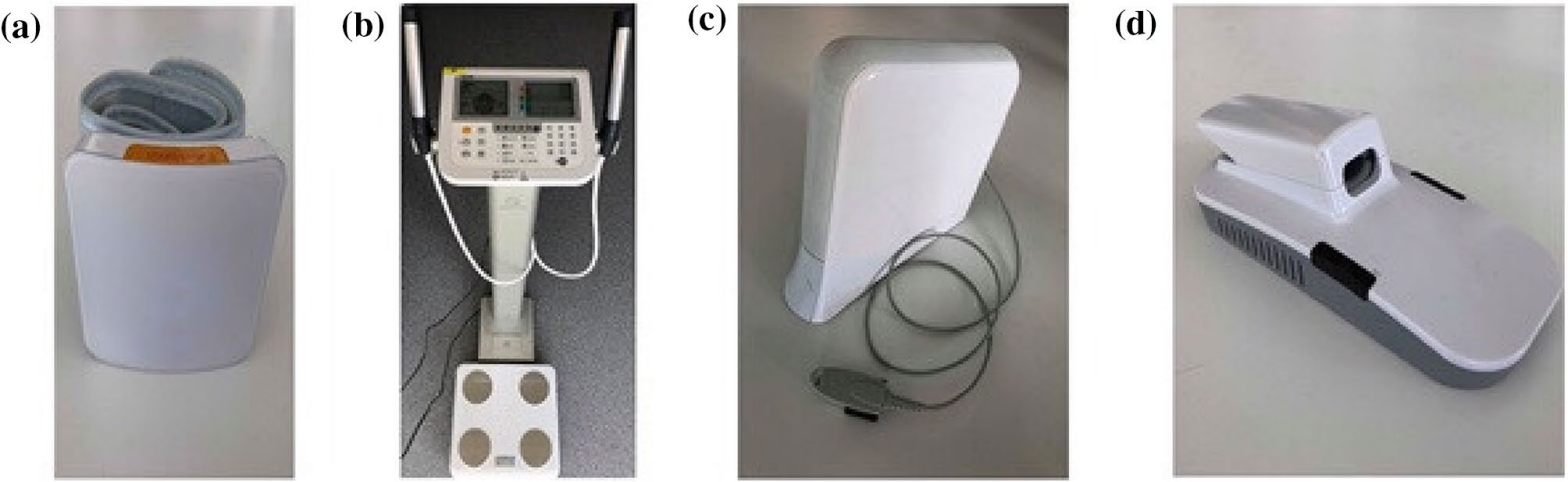
Non-invasive devices (**a**) blood pressure monitor iHealth Sense, (**b**) bioimpedance-meter Tanita MC780MA, (**c**) cardiovascular diagnostic complex AngioScan-01, (**d**) BG20 blood glucose meter.

First, a unique biomedical testbed based on non-invasive devices was designed. In order to develop appropriately tailored health tests for participants from Lindängen, specific devices were used, which were less intrusive compared to conventional devices. For instance, blood pressure was measured on the wrist instead of the upper arm, Fig. 1a. Measurements of body composition were performed using bioelectrical impedance analysis instead of using tape measurements, thus limiting direct body contact to a minimum, Fig. 1b. Additional non-invasive devices were also included in the health test, viz. the cardiovascular diagnostic complex AngioScan-01, Fig. 1c, and the non-invasive blood analyzer, BG20, Fig. 1d, to achieve a comprehensive non-invasive biomedical test.

### Participants

For biomedical testing 56 volunteers from Lindängen were identified. 17 of these participants did not fully complete the biomedical tests and were therefore excluded from data analysis. Thus, a total of 39 female volunteers, aged 25–77 years, participated in this study (Table 1). Participants were first- or second-generation migrants predominantly of Middle Eastern origin. A third of the participants were unemployed and had low education levels. They had higher risks for chronic diseases such as diabetes and heart diseases and also experienced difficulties to access health care owing to language barriers^19^.

**Table 1:** Basic characteristics of participants.

Characteristics	Participants (*n* = 39)
Age range (md)	25–77 (47)
**Educational qualification**	
University education (≥ 3 years)	7 (18%)
University education (< 3 years)	2 (5%)
Adult education at high school level	1 (3%)
High school (3–4 years)	7 (18%)
High school (2 years)	7(18%)
Elementary school	15 (38%)
**Occupational status**	
Employed	3 (8%)
Self-employed	1 (3%)
Parental leave	1 (3%)
Studying/Internship	14 (36%)
Prolonged sick leave	2 (5%)
Retired	6 (15%)
Unemployed	6 (15%)
Homemaker	6 (15%)

Three health promoters were involved in contacting citizens from the neighborhood using information leaflets providing a description on the purpose of the study and the different time points the participants were to be gathered to respond to the surveys and the biomedical tests. The health promoters also placed flyers in the municipality premises including meeting places and in the neighborhoods center and also dispersed information through social media channels as WhatsApp. The vocal communication was predominantly in Arabic language.

### Administration of health outcomes

The participants were instructed to remain calm and quite before and during the biomedical measurements. The participants were not asked to fast prior to the measurements. The attendance rate exceeded 95% during all sessions, both when the surveys were administered, and when biomedical measurements were performed. The participants also received feedback on their health outcomes in their mother tongue, most often Arabic, by the trained biomedical analysists who performed the data collection. A health promoter, *i.e.*, a representative from the community, acted as session support^19^.

### Biomedical measurements

Systolic and diastolic blood pressures (SBP and DBP, respectively) and resting heart rate (RHR) were measured using an iHealth Sense wireless wrist monitor from iHealth Labs Inc. (Sunnyvale, California, USA). The monitor was placed on the participants’ wrist.

Bioelectrical impedance analysis was performed using a bioimpedancometer MC780MA from Tanita (Tokyo, Japan). This analyzer differentiates between the individuals’ fat mass (FM), muscle mass (MM) and water mass, as well as automatically calculates body mass index (BMI) and metabolic age. The participants were instructed to stand on the device with bare feet placed on the electrode platform until their body weight was displayed. Then, participants were instructed to hold the handlebars with their arms straight down until the remaining parameters were displayed. Prior to the analysis, the participants’ basic parameters (gender, height and body type) were entered into the memory of the device.

Vascular age (VA) and stress index (SI) were determined using an AngioScan-01 from AngioScan-Electronics (Moscow, Russia). The device was placed on the participants’ right index finger.

Glu and Hb concentrations in blood were determined non-invasively using a BG20 blood glucose meter from Yones Toptech (Shenzhen, China). The device was placed on the participants’ right index finger, and the time interval during which the participant had consumed food most recently was entered.

### Health survey

The WHO (World Health Organization) QoL-BREF survey was used to gather information regarding subjective health perceptions within the participant group^22^. The validity and reliability of the survey has been extensively tested internationally and is available in 20 different languages including Swedish^22^ and Arabic^23^. The survey contains twenty-six questions and measure four broader health areas, *i.e.*, physical health, mental health, social relations and environmental health domains, as well as two global items, one is Health related QoL and the second is Health satisfaction (vide infra). A raw score of each domain and item can be calculated and transferred into range between 0–100 and 0–5, respectively. Higher scores denote higher health-related QoL^22^.

### Data analysis

Relationships between self-reported health measures and biomedical measures were analyzed using univariate (nonparametric correlation statistics) and multivariate (parametric correlation statistics) regression analyses employing software packages SPSS Statistics from IBM Corp. (Armonk, New York, USA) and Wolfram Mathematica software package from Wolfram Research (Champaign, Illinois, USA), respectively. The default significance level in all calculations was 0.05. During multivariate regression analysis models were built using all data points acquired in the investigation. An initial model was based on all variables and was further improved by discarding variables or adding nonlinearities. All statistical properties of models and residuals are described alongside with the results.

At the beginning, in absence of a relevant model, nonparametric correlation statistics was exploited, specifically Spearman’s and Kendal’s rank tests were carried out. Later, multivariate regression analysis was performed. The identification of variables, which statistically significantly correlate (*p*-value < 0.05) was carried out using a meta-modelling approach. Since linear multi-variable models failed to provide any statistically significant correlations, a 2-term set of all possible models with inclusion of linear, inverted, squared, and pairwise multiplied variables was investigated to identify statistically important variables. A set of identified statistically significant variables, which are presented in Table 2, was used to build all possible 4-term models, which were tested, and the best model was selected for each case. Specifically, all the models were firstly sorted based on the maximum *p*-value for each parameter of the model. Secondly, the models, which passed *p*-value 0.05 threshold were sorted by the sum of residuals. Finally, the best model among the models with *p*-values below 0.05 for all parameters was selected according to the sum of model residuals.

**Table 2:** Variables used in meta-modeling approach.

Full name	Abbreviation
Age	age
Systolic blood pressure	SBP
Diastolic blood pressure	DBP
Resting heart rate	RHR
Fat mass	FM
Muscle mass	MM
Body mass index	BMI
Vascular age	VA
Stress index	SI
Hemoglobin	Hb
Glucose	Glu

### Ethics approval, consent to participate and for publication

Prior to initiation of the study, the Regional Ethical Committee in Lund approved this study (DNR 2018-384 and DNR 2019-01741) based on the written ethical application prepared in accordance with the World Medical Association Declaration of Helsinki ”Ethical Principles for Medical Research Involving Human Subjects”. All activities were performed in accordance with guidelines and regulations provided by the Regional Ethical Committee. All participants were initially informed that they will take part in a research process, the results of which might be published. They were also informed that the purpose of the research was to investigate new technologies in their area, Lindängen, Malmö, Sweden. Participation was voluntary and participants were informed that they could leave the study at any time. Written information on the research process was also provided and participants were asked to sign an informed consent sheet. All materials collected were marked by code and kept confidential and shall be accessed only by the research team members.

## Results

^19^Biomedical tests revealed that 25.6% (*n* = 10) of the participants had blood pressures outside of the reference interval, Supplementary Fig. S1(a) and Fig. S1(b), whereas only one participant (2.6%) had a RHR outside of the reference interval, Fig. S1(c). The reference intervals for SBP and DBP, as well as RHRs, were defined according to Refs.^24,25^.

In accordance with the literature, overweight and obesity are defined by BMIs of ≥ 25 kg/m^2^ and ≥ 30 kg/m^2^, respectively^26^. Thus, 25.6% (*n* = 10) of the participants were considered to be overweight and 51.3% (*n* = 20) were obese, Supplementary Fig S2. More than half of the participants had body fat percentages above the reference intervals, Supplementary Figs. S3(a)-3(c), *i.e.* 21–33% of body fat for 20–39 year olds, 24–34% of body fat for 40–59 year olds, and 25–36% of body fat for 60–79 year old individuals^27^.

VA and SI were also determined based on RHR, stiffness of blood vessels, and differences of arterial pressure^28^ using the professional dual-channel cardiovascular complex, Supplementary Fig. S4. According to the manufacturer, the reference interval for the stress index is 0–150 units, whereas VA should be equal to or below the age of the participant^28^. It was found that many volunteers had both parameters above the cut-off values, Supplementary Fig. S4. Moreover, Hb and Glu concentrations in blood were determined non-invasively, Supplementary Fig. S5. Interestingly, almost all obtained values were within the reference intervals, 120–160 g/L for Hb and 2.8–11.1 mM/L for Glu^29^.

In parallel, the QoL-BREF survey was distributed among the participants. Table 3 shows the obtained QoL scores. All the mean domain scores were around the fifty-percentage mark, meaning that the sample were distributed in relative equal proportions around the cut-off level for satisfaction in QoL. However, about half of the participants (*n* = 17) had domain scores lower than the satisfactory level.

**Table 3:** QoL scores of participants.

WHOQOL-BREF score (scale)	Mean score (SD)	Score range
Physical health domain	54.3 (19.3)	0 – 100
Psychological domain	54.0 (15.9)	0 – 100
Social relationships domain	63.8 (18.8)	0 – 100
Environmental domain	56.0 (16.7)	0 – 100
Health related quality of life global item	3.5 (0.9)	0 – 5
Health satisfaction global item	3.9 (1.1)	0 – 5

Certain medical conditions of the participants from Lindängen were directly compared to the general Swedish population (Table 4). The overweight incidence was remarkably lower for the participants from Lindängen, compared to the Swedish population^30^, whereas Lindängen’s participants had a higher incidence of obesity. According to the literature, hypertension is defined by a SBP of ≥ 140 mmHg or DBP of ≥ 90 mmHg^31^. Taken these values into account one could conclude that the level of hypertension among the participants was slightly higher compared to the Swedish population^32^.

**Table 4:** Comparison of medical conditions in Lindängen to the general Swedish population.

	General Swedish population (%)	Lindängen, Malmö (%) (*n*)
Overweight	52.4	25.6 (*n* = 10)
Obesity	20.4	51.3 (*n* = 20)
Hypertension	22.3	25.6 (*n* = 10)

Last but not least, detailed statistical analysis of the data was performed. Spearman’s and Kendal’s rank tests revealed no significant correlation between the four QoL domain scores/two of the global items and biomedical metrics. This was unsurprising since the dependence between perceived health and biomedical metrics is a priori complex. In other words, it is inappropriate to infer strong linear dependences between participant-perceived QoL scores and individual biomedical parameters, such as body composition parameters, RHR, blood pressure, etc., using a univariate regression analysis. Thus, multivariate regression analysis of all the data collected was carried out, as described in the Method section, and the final results are presented below.

The social relationships domain and health related QoL global item scores have no statistically significant correlations with variables. The best model found to describe physical health domain scores is presented in Eq. 1, while the statistical properties of the model are provided in ANOVA Table 5.

1 $$ - 22.2452 + 1799.6/{\text{BMI}} + 0.0157277 \cdot {\text{FM}} \cdot {\text{VA}} - 0.000567556 \cdot {\text{VA}} \cdot {\text{SI}}$$

**Table 5:** The statistical properties of the best model for physical health domain scores.

	DF*	SS*	MS*	F-statistic	*p*-value
1/BMI	1	1539.19	1539.19	5.26575	0.0278616
FM^.^VA	1	1234.2	1234.2	4.22235	0.0474191
VA^.^SI	1	1287.95	1287.95	4.40625	0.0430892
Error	35	10,230.6			
Total	38	14,291.9			

*Here and below: DF – degree of freedom, SS – sum of squares; MS – mean squares.

As one can see from Table 5, all coefficients are statistically significant. On the one hand, the model shows that larger BMI lowers expected physical health domain scores. On the other hand, surprisingly, larger FM and VA, rise physical health domain scores. However, it should be noted that there is a linear correlation between variables FM and BMI, and hence it may be a manifestation of the approximate quadratic dependence on BMI. The SI in synergy with VA lower physical health domain scores. The physical health domain scores predicted by the model have a 0.533 statistically significant correlation coefficient with a *p*-value of 0.00048.

The best model found to describe psychological domain scores is presented in Eq. 2, while the statistical properties of the model are provided in ANOVA Table 6.

2 $$- 20.2236 - 1092.97/{\text{age}} + 2257.35/{\text{BMI}} + 0.0218865 \cdot {\text{FM}} \cdot {\text{BMI}}$$

**Table 6:** The statistical properties of the best model for psychological domain scores.

	DF	SS	MS	F-statistic	*p*-value
1/age	1	1641.41	1641.41	9.44392	0.00408733
1/BMI	1	1067.05	1067.05	6.13935	0.0181931
FM^.^BMI	1	822.234	822.234	4.73077	0.0364706
Error	35	6083.2			
Total	38	9613.9			

As one can see from Table 6, all coefficients are statistically significant. Age tends to lower psychological domain scores, however, with increasing age, the negative impact on psychological domain scores is attenuated. Psychological domain scores are increased by smaller BMIs and by a synergistic interaction of BMI with FM. The psychological domain scores predicted by the model have a 0.606 statistically significant correlation coefficient with a *p*-value of 0.000043.

The best model found to describe environmental domain scores is presented in Eq. 3, while the statistical properties of the model are provided in ANOVA Table 7.

3 $$ 70.5533 - \left( {8.39801 \cdot {\text{RHR}}} \right)/{\text{age}}$$

**Table 7:** The statistical properties of the best model for environmental domain scores.

	DF	SS	MS	F-statistic	*p*-value
RHR/age	1	1099.94	1099.94	4.2593	0.0461023
Error	37	9555.04			
Total	38	10,655.00			

As one can see from Table 7, the coefficient is statistically significant. Age tends to inversely lower environmental domain scores and the effect is attenuated with age and increases with RHR. The environmental domain scores predicted by the model have a weakly statistically significant correlation coefficient of 0.32 with a *p*-value of 0.046.

The best model found to describe health satisfaction is presented in Eq. 4, while the statistical properties of the model are provided in ANOVA Table 8.

4 $$ - 2.73262 + 84.1044/{\text{FM}} + 0.000445315\;{\text{age}} \cdot {\text{FM}} + 0.000449529\;{\text{SBP}} \cdot {\text{FM}}$$

**Table 8:** The statistical properties of the best model for health satisfaction global item scores.

	DF	SS	MS	F-statistic	*p*-value
1/FM	1	8.68201	8.68201	10.3839	0.00274869
Age^.^FM	1	5.24192	5.24192	6.2695	
SBP^.^FM	1	3.58184	3.58184	4.28398	0.0459167
Error	35	29.2635			
Total	38	46.7692			

As one can see from Table 8, all calculated coefficients are statistically significant. FM has an inverse positive effect on health satisfaction scores, which are synergistically weakly affected by age and SBP. The health satisfaction scores predicted by the model have a 0.612 statistically significant correlation coefficient with a *p*-value of 0.000035.

## Discussion

First, the unique mobile testbed based on non-invasive technologies developed in the current studies was considered to be less intrusive compared to conventional methods due to minimizing of physical contact and exposure of skin, which was beneficial for individuals with specific backgrounds *e.g.*, from the Middle East. Moreover, portability allowed us to perform the test in Lindängen, at the convenience of the participants and most importantly free of cost as desired by the participants themselves.

Second, biomedical studies revealed high SIs measured in more than 50% of the volunteers, which is an extreme situation for an average apparently healthy population. On the one hand, this is not surprising since lower socioeconomic status has been linked to long-term stress, which can manifest in individuals as physiological stress^33^. Moreover, it is believed that low socioeconomic status harms health through dysregulation of the physiological stress response systems^34^. On the other hand, one of the strong indicators of a high stress level with a high risk of chronic diseases is higher RHR. Several studies have reported on the association between elevated RHR and cardiovascular mortality^35,36^. However, in our studies almost all participants had a RHR within the reference interval, Supplementary Fig. S1(c), and their vascular age was scattered, in comparison with their real age, Supplementary Fig. S4(a), which is expected in an on average apparently healthy population. It should be emphasized that even though non-invasive determination of Glu and Hb concentrations in blood were included in biomedical analyzes, due to the current lack of evidence regarding the ability of the device to determine these bioanalytes accurately, the results from non-invasive measurements should be treated with cautions, Supplementary Figs. S5(a) & S5(b).

Third, when comparing certain medical conditions of the participants from Lindängen to the general Swedish population (Table 4),^30–32^surprisingly, the mean scores of psychological and physical health domains were very close to each other despite of high SI measured in more than 50% of the volunteers. Moreover, significant correlation between psychological domain scores and SI was not observed, whereas the SI in synergy with VA lowered physical health domain scores.

As stated above, the participants from Lindängen were a uniform group of female volunteers from the Middle East. On the one hand and to some extent, the ethnicity of the participants represents the area due to the high number of residents in Malmö who are not born in Sweden^37^. On the other hand, the participants were not ideal representatives of the area due to the uniformity in gender and old age bias. The ratio between female and male residents in Malmö is *ca.* 50/50^37^. Moreover, almost half of the residents in Malmö are under 35 years, whereas the average age among the participants was 49 years (Table 1). Thus, one should consider that biomedical conclusions made in the current studies could possibly understate the general health status of people in Lindängen. In other words, the small sample from this socioeconomically disadvantaged community makes the interpretations from Table 4 more indicative than conclusive.

Last but not least, comprehensive statistical analysis of all the results was carried out. To the best of our knowledge, this is the first study in the field of CBPR, where multivariate non-linear regression analysis of health outcomes was exploited. Many interesting correlations between perceived health-related QoL and biomedical metrics were disclosed, *i.e.*, between physical health domain scores, BMI, FM, and VA; psychological domain, age, BMIs, and FM; environmental domain, age and RHR; health satisfaction global item, FM, age, and SBP. It can be concluded that strong relationships between perceived health and biomedical metrics do exist, and even though they are very complex, the recognition of which is essential. Specifically, discovered relationships between BMI, RHR, SBP, etc., well-known markers for chronic diseases, and self-reported QoL measures call for targeted interventions, which take into consideration not only biological but also perceived influence of social and psychological factors, to achieve increased health-equity.

The social relationships domain and self-reported QoL scores have no statistically significant correlations with variables. However, it should be noted, that all models were found from a limited subset of variables and more interesting correlations could be disclosed by expanding the number of terms used in the evaluation of the models. This, of course, is computationally costly, and the model space increases factorially with the number of terms included in a model.

Studies exploring the relationships between comprehensive biomedical assessments and self-reported WHO QoL-BREF scores are few and far between. Previous investigations have only examined the relationships between measurements of blood pressure or BMI in general population groups using univariate regression analysis^7,38–40^. Nevertheless, our results are somehow in line with a previous study describing a relationship between BMI and general health satisfaction, where the strength of the correlation coefficient particularly among older women was low (*r* = 0.025)^7,38–40^. According to earlier studies, women below 55 years of age are more concerned about their body weight, BMI, and body image compared to older women. The current study included women with a median age of 47 years, which may explain the stronger correlation between health satisfaction and body composition in this group of women^41^. Most participants in this study singled out weight loss as a motivation to participate in the physical activity intervention that was to follow the biomedical assessments. Participants seemed to be more aware of the significance of body weight in relation to their health, in contrast to the lack of understanding of the other complex measurements performed in this study. Such an awareness can be tapped in future health promotional efforts to socioeconomically disadvantaged groups to address obesity and overweight related problems.

The implementation of the non-invasive testbed in Lindängen was highly appreciated by the participants, as indicated by the high attendance rate in the data gathering process. The participants realized that the different health parameters tested could easily be used to monitor their health in relation to the risks of developing different diseases including cardiovascular disorders. Also, participants were excited about the health tests since they expressed a lack of trust in the health system in general in Sweden, and had no wish to be analyzed using conventional setups. Since they had been offered the time and personal contact when being tested, and were also given the opportunity to learn the significance of measured health parameters using their native language, the participants appreciated the health test even more. Taking these findings in consideration with the results from the current work, the evidence of using non-invasive health tests for people in a socioeconomically challenged suburb in Sweden seems to be supportive from several perspectives in relation to evaluating health descriptors.

The additional findings, when comparing participant results with generic health data from Sweden, also indicate that health is to be considered as a multifaceted phenomenon that needs to be approached using several perspectives and data gathering methods. Given the evidence from this study, health should never be summarized or evaluated relying on a single circumstance, as this may lead to very different conclusions regarding the overall participant health status. Rather, a more elaborate approach using both objective and subjective health parameters, as well as qualitative data targeting health related descriptors, is preferable to gauge and understand the complex phenomenon of health in these communities.

## Conclusions

The uniquely designed testbed based on non-invasive assessment tools, accompanied with the WHO QoL survey, was implemented in a socioeconomically disadvantaged community. The application of the testbed in Lindängen was highly appreciated by the participants. The findings of the current study illustrate that the physical health of the participants from the ethnically diverse low-income neighborhood of Malmo, as evident from biomedical parameters, was comparable to the general Swedish population, apart from a higher number of obese individuals and fewer cases of overweight. Using multivariate non-linear regression analysis interesting correlations between perceived health and biomedical metrics were disclosed. The results from this study strongly suggest the need to assess health using multiplex methods along with different mathematical/statistical approaches, especially in socially disadvantaged communities. Thus, future efforts should be devoted towards the automatization of data gathering process, proper data handling and storage following current General Data Protection Regulation rules, as well as advance analysis of the collected data using different mathematical/statistical approaches, including the development and exploitation of advanced Artificial Intelligence based diagnostic systems.

## Supplementary Information

Supplementary Information.

## Data Availability

The datasets used and/or analyzed during the current study are available from the corresponding author on reasonable request.

## Abbreviations

BMI: Body mass index
CBPR: Community-based participatory research
DBP: Diastolic blood pressure
DF: Degree of freedom
FM: Fat mass
Glu: Glucose
Hb: Hemoglobin
MM: Muscle mass
MS: Mean squares
QoL: Quality of life
RHR: Resting heart rate
SBP: Systolic blood pressure
SI: Stress index
SS: Sum of squares
VA: Vascular age
WHO: World Health Organization

## References

[1] Yamani, A. Z. *et al.* A proposed noninvasive point-of-care technique for measuring hemoglobin concentration. In: Proceedings of the 2019 International Conference on Computer Information Science 487–490 (2019).

[2] Howell JD (1991). Diagnostic technologies: X-rays, electrocardiograms, and cat-scans. South. Calif. Law Rev..

[3] Fye WB (1994). A history of the origin, evolution and impact of electrocardiography. Am. J. Cardiol..

[4] Bonetti PO (2004). Noninvasive identification of patients with early coronary atherosclerosis by assessment of digital reactive hyperemia. J. Am. Coll. Cardiol..

[5] Lukaski HC, Johnson PE, Bolonchuk WW, Lykken GI (1985). Assessment of fat-free mass using bioelectrical impedance measurements of the human body. Am. J. Clin. Nutr..

[6] Khalil SF, Mohktar MS, Ibrahim F (2014). The theory and fundamentals of bioimpedance analysis in clinical status monitoring and diagnosis of diseases. Sensors.

[7] Barker SJ, Shander A, Ramsay MA (2016). Continuous noninvasive hemoglobin monitoring: A measured response to a critical review. Anesth. Analg..

[8] Taylor MB, Whitwam JG (1986). The current status of pulse oximetry - Clinical value of continuous noninvasive oxygen-saturation monitoring. Anaesthesia.

[9] Tierney MJ (2001). Clinical evaluation of the GlucoWatch (R) biographer: a continual, non-invasive glucose monitor for patients with diabetes. Biosens. Bioelectron..

[10] Caduff A (2006). Non-invasive glucose monitoring in patients with diabetes: A novel system based on impedance spectroscopy. Biosens. Bioelectron..

[11] Smith, J. L. The pursuit of noninvasive glucose: "Hunting the deceitful Turkey". Seventh Edition. NIVG Consulting, LLC, Portland, OR, USA, 244 pp. (2006).

[12] Ahrens T, Tucker K (1999). Pulse oximetry.

[13] Welfare, A. I. O. H. A. Health across socioeconomic groups. *Australia’s Health* **15** (2016).

[14] Melander D (2020). Diagnostics as the key to advances in global health: Proposed methods for making reliable diagnostics widely available. J. Med. Device.

[15] Nationella Operative Avdelningen, "Utsatta områden - Social ordning, kriminell struktur och utmaningar för polisen". https://polisen.se/siteassets/dokument/ovriga_rapporter/utsatta-omraden-social-ordning-kriminell-struktur-och-utmaningar-for-polisen-2017.pdf (2017).

[16] Avery, H., Sjogren Forss, K. & Ramgard, M. Empowering communities with health promotion labs: result from a CBPR programme in Malmo, Sweden. *Health Promot. Int.* 1–15 (2021). 10.1093/heapro/daab069 PMC8851348 34263320

[17] Israel, B. A., Eng, E., Schulz, A. J. & Parker, E. A. Introduction to methods in community-based participatory research for health 1st edition. Jossey-Bass 524 pp. (2005).

[18] collaborative non-linear processes of knowledge mobilization (2017). Abma Tineke, A. *et al.* Social impact of participatory health research. Educ. Action Res..

[19] Ramji R (2020). Development and evaluation of a physical activity informed by participatory research- a feasibility study. BMC Public Health.

[20] World Health Organization. *Towards a common language for Functioning, Disability and Health: ICF*. Geneva, 1–22 (2002).

[21] World Health Organization. *Constitution of the world health organization*. Geneva, 1-18 (1995).

[22] Harper A, Power M, Grp W (1998). Development of the World Health Organization WHOQOL-BREF quality of life assessment. Psychol. Med..

[23] Ohaeri JU, Awadalla AW (2009). The reliability and validity of the short version of the WHO quality of life instrument in an Arab general population. Ann. Saudi Med..

[24] Carretero OA, Oparil S (2000). Essential hypertension Part I: Definition and etiology. Circulation.

[25] Cook STM, Schaub MC, Wenaweser P, Hess OM (2006). High heart rate: a cardiovascular risk factor?. Eur. Heart J..

[26] Crunelle CL (2017). Influence of body mass index on hair ethyl glucuronide concentrations. Alcohol Alcohol..

[27] Total Row Fitness. *Healthy body fat ranges for adults.* https://totalrowfitness.com/know-your-body-fat-total-row-explains-why-it-matters (2018).

[28] Angioscan Electronics LLC. AngioScan-01P, AngioScan-01M: Personal device for cardiovascular system diagnosis. Moscow, Russia 128 pp. (2014).

[29] Cheng CK, Chan J, Cembrowski GS, Van Assendelft OW (2004). Complete blood count reference interval diagrams derived from NHANES III: Stratification by age, sex, and race. Lab. Hematol..

[30] World Health Organization. *Sweden - Diabetes country profiles*. Geneva (2016).

[31] DeJongste, M. J. L. *Hypertension (high blood pressure).* https://www.neuromodulation.com/hypertension (2012).

[32] World Health Organization. *Global Health Observatory data repository - Raised blood pressure (SBP ≥ 140 OR DBP ≥ 90), crude (%)*. Geneva (2015).

[33] Zhang, A., et al. Resting heart rate, physiological stress and disadvantage in Aboriginal and Torres Strait Islander Australians: analysis from a cross-sectional study. *BMC Cardiovas. Disord.* **16**, 36/31–36/38 (2016). 10.1186/s12872-016-0211-9 PMC4751751 26868922

[34] Le-Scherban F (2018). Child and adult socioeconomic status and the cortisol response to acute stress: Evidence from the multi-ethnic study of atherosclerosis. Psychosom. Med..

[35] Palatini P, Jullius S (2004). Elevated heart rate: A major risk factor for cardiovascular disease. Clin. Exp. Hypertens..

[36] Palatini P, Benetos A, Julius S (2006). Impact of increased heart rate on clinical outcomes in hypertension - Implications for antihypertensive drug therapy. Drugs.

[37] Malmö Stad, M. *Statistik för Malmös områden.* https://malmo.se/Fakta-och-statistik/Statistik-for-Malmos-omraden.html (2018).

[38] Chung PK, Zhao YN, Liu JD, Quach B (2017). A canonical correlation analysis on the relationship between functional fitness and health-related quality of life in older adults. Arch. Gerontol. Geriatr..

[39] Herman KM, Hopman WM, Rosenberg MW (2013). Self-rated health and life satisfaction among Canadian adults: associations of perceived weight status versus BMI. Qual. Life Res..

[40] Neumark-Sztainer D, Paxton SJ, Hannan PJ, Haines J, Story M (2006). Does body satisfaction matter? Five-year longitudinal associations between body satisfaction and health behaviors in adolescent females and males. J. Adolescent Health.

[41] Pruis TA, Janowsky JS (2010). Assessment of body image in younger and older women. J. General Psychol..

