# Supplementary material for: Developing and evaluating non-invasive healthcare technologies for a group of female participants from a socioeconomically disadvantaged area
Source: Sci Rep. 2021 Dec 13;11:23896. doi: 10.1038/s41598-021-03262-3 (PMC8668900; doi:10.1038/s41598-021-03262-3)
Supplement: Supplementary file 1 — Supplementary Information. [file 41598_2021_3262_MOESM1_ESM.docx]

**Developing and Evaluating Non-invasive Healthcare Technologies for a Group of Female Participants from a Socioeconomically Disadvantaged Area**

**Eman Awad^1^, Rathi Ramji^2^, Stefan Cirovic^1^, Margareta Rämgård^2^, Anders Kottorp^2^, Sergey Shleev^1*^**

^1^ Department of Biomedical Science, Health and Society and Biofilms-Research Center for Biointerfaces, Malmö University, 20560 Malmö, Sweden

^2^ Department of Care Science, Health and Society, Malmö University, 20560 Malmö, Sweden

**^*^**Corresponding author:

Sergey Shleev

[sergey.shleev@mau.se](mailto:sergey.shleev@mau.se)

**Supplementary material**


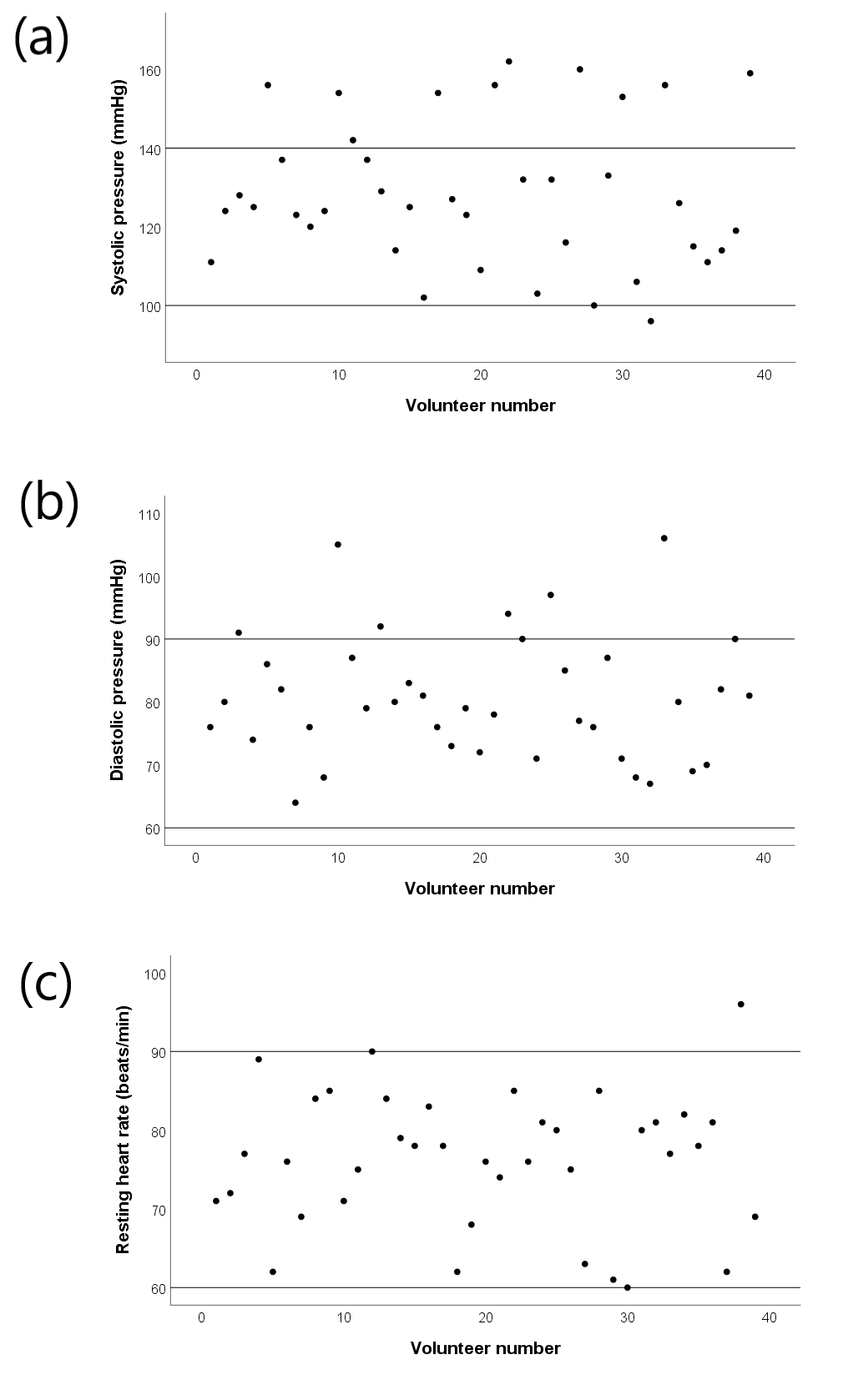


**Supplementary Fig. S1 Blood pressures and resting heart rates. (a) systolic pressures, (b) diastolic pressures, and (c) resting heart rates.**


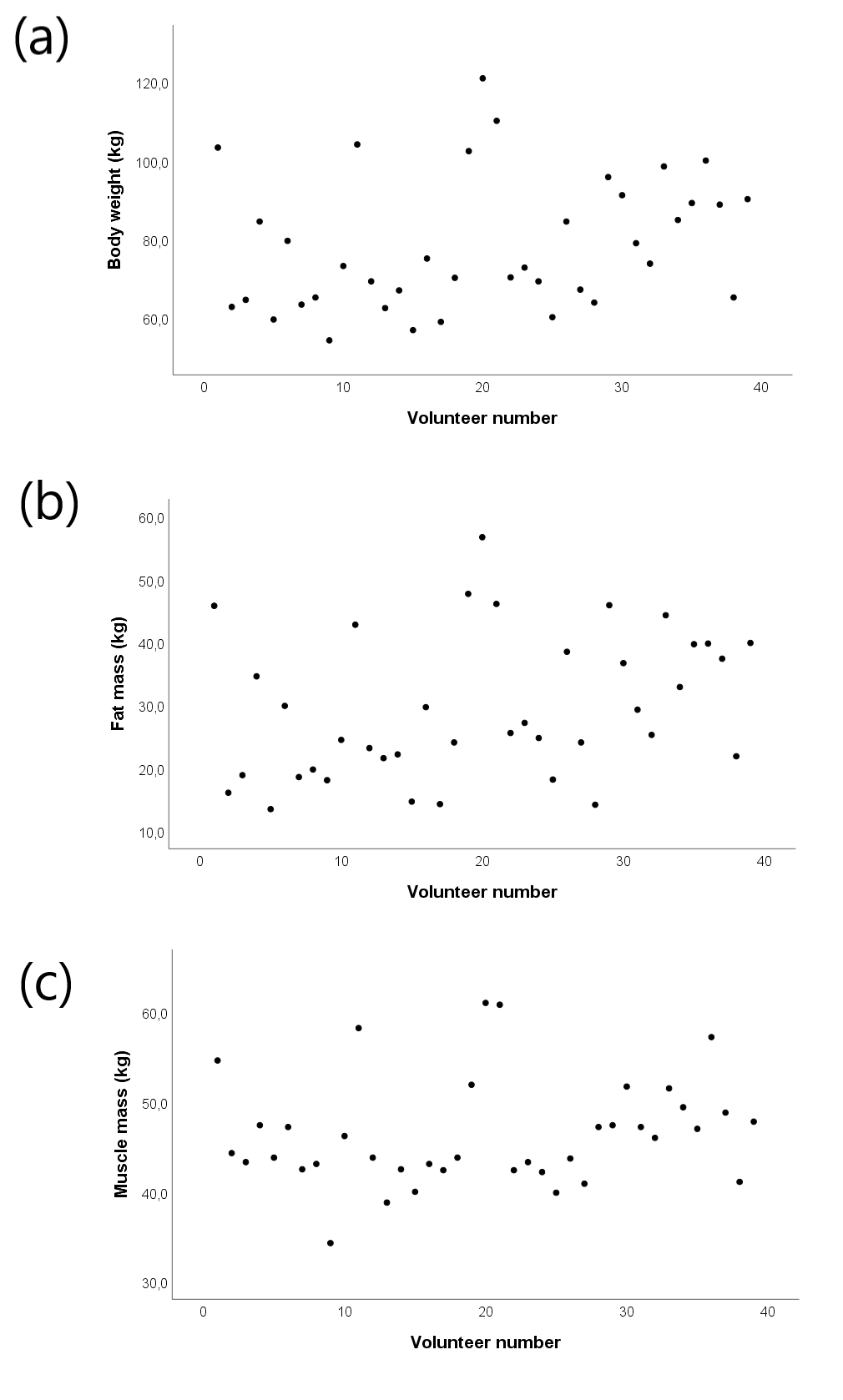


*
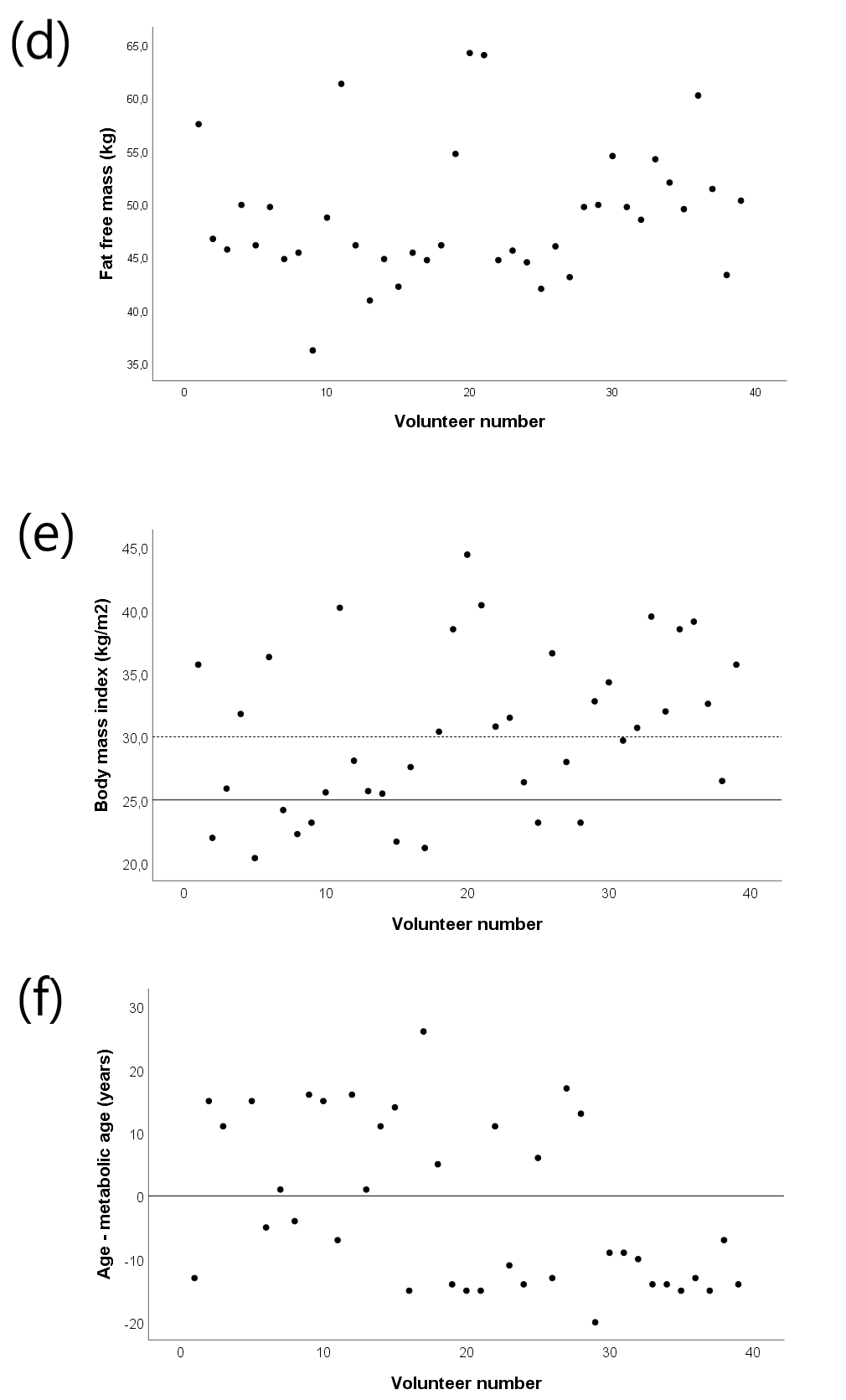
*

**Supplementary Fig. S2 Body composition analysis (a) body weight, (b) fat mass, (c) muscle mass, (d) fat free mass, (e) body mass indexes, and (f) difference in real and metabolic ages.**


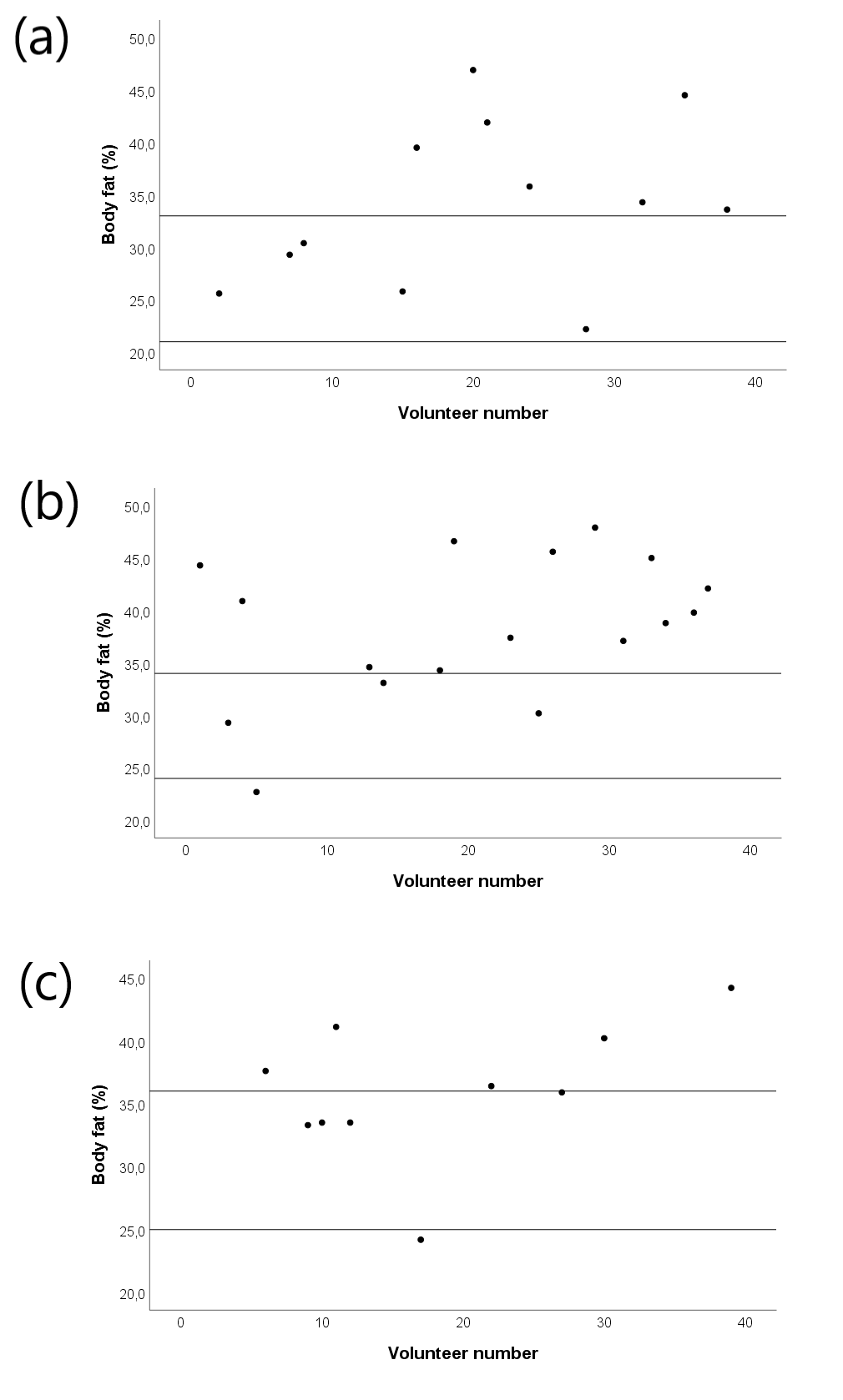


**Supplementary Fig. S3 Body composition analysis. (a) body fat in the age group 20-39 years, (b) body fat in the age group 40-59 years, and (c) body fat in the age group 60-79 years.**


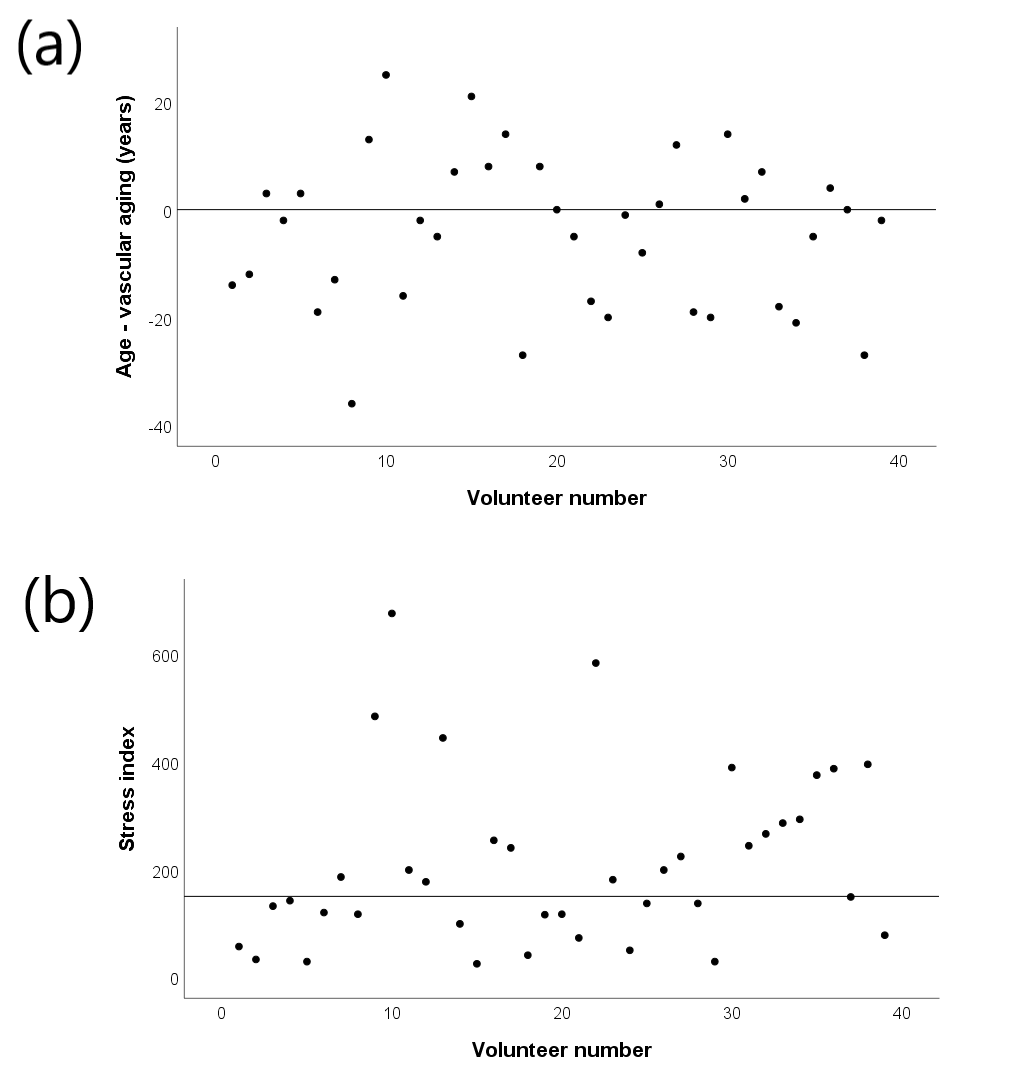


**Supplementary Fig. S4 Vascular system analysis (a) difference in real age and vascular aging, and (b) stress index.**


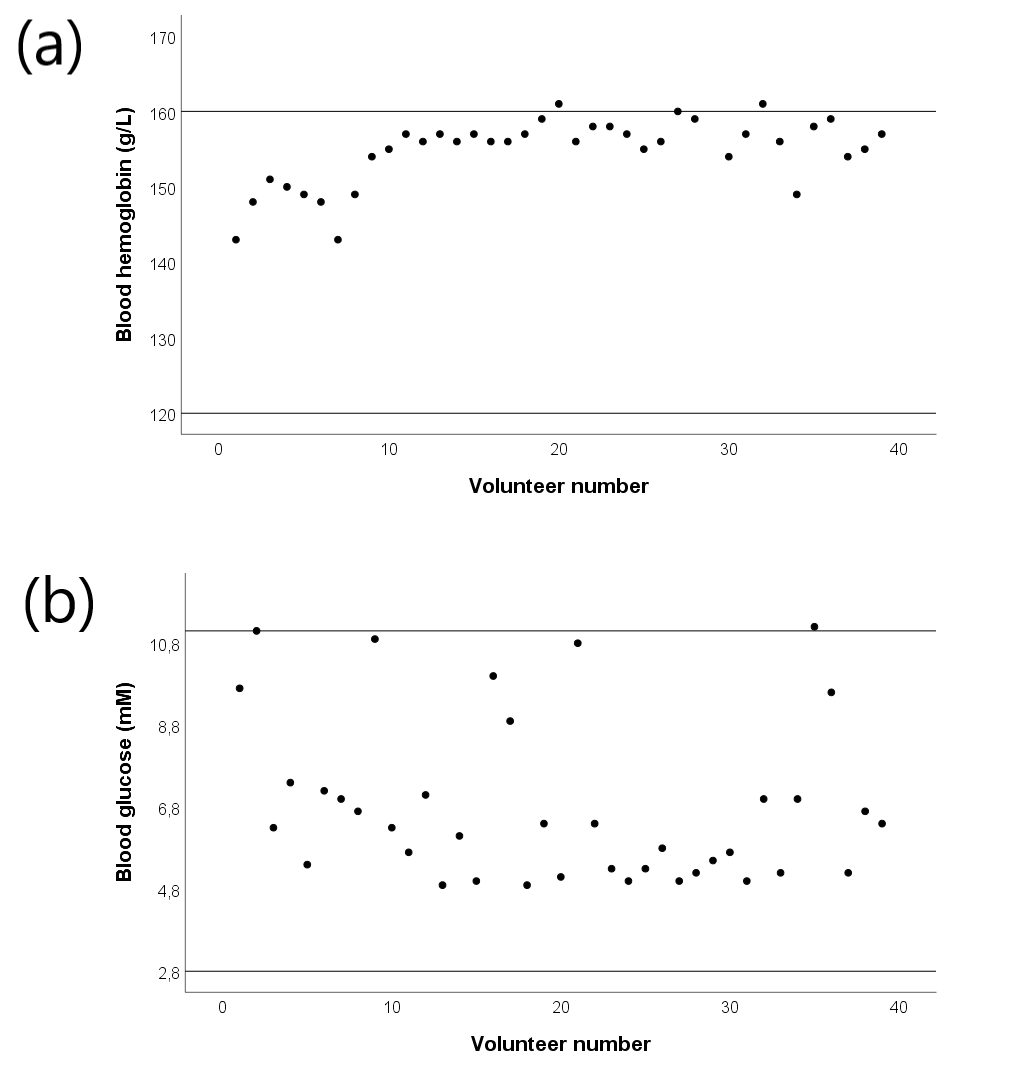


**Supplementary Fig. S5 Non-invasive determination of blood bioanalytes. (a) blood hemoglobin, (b) blood glucose.**
